# Multiscale simulations reveal TDP-43 molecular-level interactions driving condensation

**DOI:** 10.1016/j.bpj.2023.10.016

**Published:** 2023-10-17

**Authors:** Helgi I. Ingólfsson, Azamat Rizuan, Xikun Liu, Priyesh Mohanty, Paulo C.T. Souza, Siewert J. Marrink, Michael T. Bowers, Jeetain Mittal, Joel Berry

**Affiliations:** 1 Physical and Life Sciences Directorate, Lawrence Livermore National Laboratory, Livermore, California; 2 Artie McFerrin Department of Chemical Engineering, Texas A&M College of Engineering, College Station, Texas; 3 Department of Chemistry & Biochemistry, University of California Santa Barbara, Santa Barbara, California; 4 Molecular Microbiology and Structural Biochemistry (MMSB, UMR 5086), CNRS & University of Lyon, Lyon, France; 5 Laboratory of Biology and Modeling of the Cell, École Normale Supérieure de Lyon, Université Claude Bernard Lyon 1, CNRS UMR 5239 and Inserm U1293, 46 Allée d’Italie, Lyon, France; 6 Groningen Biomolecular Science and Biotechnology Institute, University of Groningen, Groningen, the Netherlands; 7 Department of Chemistry, Texas A&M University, College Station, Texas; 8 Interdisciplinary Graduate Program in Genetics and Genomics, Texas A&M University, College Station, Texas

## Abstract

The RNA-binding protein TDP-43 is associated with mRNA processing and transport from the nucleus to the cytoplasm. TDP-43 localizes in the nucleus as well as accumulating in cytoplasmic condensates such as stress granules. Aggregation and formation of amyloid-like fibrils of cytoplasmic TDP-43 are hallmarks of numerous neurodegenerative diseases, most strikingly present in >90% of amyotrophic lateral sclerosis (ALS) patients. If excessive accumulation of cytoplasmic TDP-43 causes, or is caused by, neurodegeneration is presently not known. In this work, we use molecular dynamics simulations at multiple resolutions to explore TDP-43 self- and cross-interaction dynamics. A full-length molecular model of TDP-43, all 414 amino acids, was constructed from select structures of the protein functional domains (N-terminal domain, and two RNA recognition motifs, RRM1 and RRM2) and modeling of disordered connecting loops and the low complexity glycine-rich C-terminus domain. All-atom CHARMM36m simulations of single TDP-43 proteins served as guides to construct a coarse-grained Martini 3 model of TDP-43. The Martini model and a coarser implicit solvent C⍺ model, optimized for disordered proteins, were subsequently used to probe TDP-43 interactions; self-interactions from single-chain full-length TDP-43 simulations, cross-interactions from simulations with two proteins and simulations with assemblies of dozens to hundreds of proteins. Our findings illustrate the utility of different modeling scales for accessing TDP-43 molecular-level interactions and suggest that TDP-43 has numerous interaction preferences or patterns, exhibiting an overall strong, but dynamic, association and driving the formation of biomolecular condensates.

## Significance

Excessive aggregation of the RNA-binding protein TDP-43 in neurons is associated with numerous neurodegenerative diseases, including amyotrophic lateral sclerosis (ALS). Determining the molecular properties of full-length TDP-43 has proven challenging as the protein aggregates in solution and a large part of the 414-amino acid protein is intrinsically disordered rendering it hard to sample using simulations. Here, we develop and utilize molecular models at multiple scales (all-atom, coarse-grained, and implicit water coarse-grained) to explore TDP-43 molecular-level interactions with itself and other TDP-43 molecules. We demonstrate the TDP-43 protein’s strong tendency to self-associate, yet doing so in a dynamic, fluid-like, manner and illustrate the utility of these different modeling scales for further studies of TDP-43.

## Introduction

Abnormal aggregation of the TAR DNA-binding protein 43 kDa (TDP-43) is associated with multiple human diseases such as amyotrophic lateral sclerosis, frontotemporal lobar degeneration, and Alzheimer’s disease (1,2,3). Under normal conditions, TDP-43 is mainly located in the cell nucleus, where it regulates the stability, translation, and splicing of mRNA, along with the production of miRNA (4,5,6). In neurodegenerative diseases, TDP-43 relocates from the nucleus to the cytoplasm where it forms insoluble inclusions that contain full-length and truncated TDP-43 proteins (7,8). Recent studies have suggested that the formation of pathogenic aggregates depends on a liquid-liquid phase separation (LLPS) mechanism (9,10,11). Cleveland and co-workers (12) showed that RNA-deficient TDP-43 can form liquid-like nuclear condensates, which are referred to as “anisosomes” in association with Hsp70 chaperones. The loss of chaperone-mediated interactions triggers a liquid-to-solid conversion which may lead to the formation of toxic aggregates.

TDP-43 is a 414 amino acid long protein composed of an N-terminal domain (NTD), two RNA recognition motifs (RRM1 and RRM2), and a C-terminal domain (CTD) (13). The NTD region is involved in reversible physiological TDP-43 self-dimerization, which is responsible for RNA splicing, phase separation, and irreversible pathological cytoplasmic aggregation (14,15,16). A few monomeric structures of the NTD region are available in the Protein Data Bank (PDB) with PDB: 5MRG (17) being the longest fragment (residues 1–102). Two RRM domains can bind sequence-specific single-stranded or double-stranded DNA/RNA and regulate RNA splicing and translation (18,19). Interestingly, RRM2 contains an amyloidogenic core that can misfold and induce native TDP-43 to form pathological aggregates responsible for neurodegenerative diseases (20,21). The three-dimensional structure of tandem RRMs bound to RNA, PDB: 4BS2 (22), is resolved. The CTD is mostly unstructured and believed to be responsible for the aberrant cytosolic aggregation of TDP-43 (23). An NMR structure (PDB: 2N3X) (24) of the amyloidogenic core is available and the transformation of this amyloidogenic core from α-helix structure to β-sheet is believed to initiate pathological aggregation (25). Owing to its large size, low solubility, and the presence of flexible linkers and a long disordered CTD (residues 261–414), the full-length TDP-43 has so far not been amenable to conventional structure determination methods such as x-ray crystallography and NMR spectroscopy. Several studies have reported on the low solubility of wild-type (WT) TDP-43 (26,27), which makes it difficult to purify in sufficient quantity for experimental characterization. Recently, Hasnain and co-workers (28) successfully purified tryptophan-free (six tryptophan to alanine), monomeric full-length TDP-43 under denaturing conditions and characterized its conformational ensemble in solution using small-angle x-ray scattering (SAXS). Overall, the SAXS-derived ensemble points toward the dynamic nature (mean R_g_ ∼4.1 nm) of monomeric TDP-43 with limited intramolecular interactions. In contrast, WT TDP-43 has a strong tendency to dimerize during in vitro purification, and in vivo dimerization can lead to higher-order assemblies through head-to-tail oligomerization (29).

The self-assembly of a multidomain protein such as TDP-43 in the context of self-oligomerization, phase separation, and aggregation involves a complex interplay between intramolecular (single-chain) and intermolecular (multichain) interactions and is strongly influenced by solution conditions such as pH and ionic strength. All-atom (AA) molecular dynamics simulations using current, state-of-the-art force fields (30) in combination with GPU accelerated algorithms are well suited to provide high-resolution details regarding the conformational dynamics and interactions of multidomain proteins at the single-chain level on the ns–*μ*s timescale. However, probing the intermolecular interactions implicated in self-assembly processes using AA simulations presents a significant computational overhead due to the large system size and timescales (ms–s) associated with these processes. Coarse-grained (CG) models, which provide a reduced representation of biomolecules, offer a viable alternative to their atomistic counterparts and allow for the investigation of self-assembly processes for large systems at significantly longer timescales. The increased speed of CG models, however, comes with a cost of lower accuracy and/or reduced domain of applicability and their limitations should always be respected (31).

A multiscale simulation approach employing both AA and CG simulations has been successfully used to identify the sequence and structural determinants of LLPS for several, disordered prion-like domains of RNA-binding proteins such as FUS, hnRNAP2, and TDP-43 (32,33,34). Notably, multiscale simulations coupled with NMR experiments showed the presence of a transient helix in the conserved region (CR) of TDP-43 CTD, which promotes its phase separation through dimerization and higher-order oligomerization (33,35). For TDP-43, much further work is needed to characterize the different contributing factors for protein assemblies and, potentially deleterious, downstream aggregation, and/or filament formation. Numerous factors, including protein structural elements, posttranslational modifications, and mutations, as well as changes in environment, have been linked to increased/decreased protein association, but how these factors combine potentially leading to pathological TDP-43 aggregation remain unclear. Here, multiscale simulations can play a crucial role linking molecular-level interactions to aggregated behavior of protein assemblies.

In this study, we utilized recent advances in molecular modeling of intrinsically disordered proteins (IDPs) and modeled full-length TDP-43 molecular-level interactions, developing and contrasting models across three scales, AA and two levels of CG models. The AA model was used to guide and verify explicit water CG model construction and explore single-protein dynamics. The explicit water CG model was used to explore TDP-43 inter- and intraprotein dynamics for several protein variants as well as initial protein assemblies. Protein assemblies were then more efficiently explored using a CG implicit water model. Combined, the models reveal some of TDP-43’s key molecular-level interactions and illustrate the utility of this type of multiscale modeling to access TDP-43 behavior.

### Simulation methods

#### Initial TDP-43 structure

A full-length TDP-43 structure was constructed using individual structures of different domains. The NTD using PDB: 5MRG (17), RRM domains using PDB: 4BS2 (22), and C-terminal helix using PDB: 2N3X (24). The missing residues were considered unstructured and built using MOE (36). Structures with and without the 12 basepair long RNA fragment bonded to both RRM1 and RRM2 in PDB: 4BS2 were considered. Missing hydrogens were added, and RNA backbone and hybridization corrected with MOE.

#### AA simulation details

TDP-43 dynamics were evaluated at the AA level with CHARMM36m (37), for which the protein force field CHARMM36 (38) has been refined to better capture IDPs. Starting structures used the following protein fragments: NTD using PDB: 5MRG, NTD in dimer structure using PDB: 6B1G (15) (with the two mutations changed back to WT), RRM domains using PDB: 4BS2 with and without RNA, C-terminal residues 311–360 using PDB: 2N3X (using Biological Assembly 1 for all fragments), and remaining residues modeled as unstructured with MOE, and full-length protein both with and without RNA. Each protein, and protein part, were set up using CHARMM-GUI solution builder (39,40) with an octahedral water box of 0.8 nm minimal distance from the protein using the CHARMM-modified TIP3P model (41) and 150 mM KCl. The CHARMM-GUI default initialization and run parameters were used. All the simulations were initialized and run eight times for 2 *μ*s each at 310.15 K using GROMACS v.2018.03 (42), with an aggregated simulation time of 144 *μ*s.

#### Explicit water CG TDP-43 models

**Figure 1:**
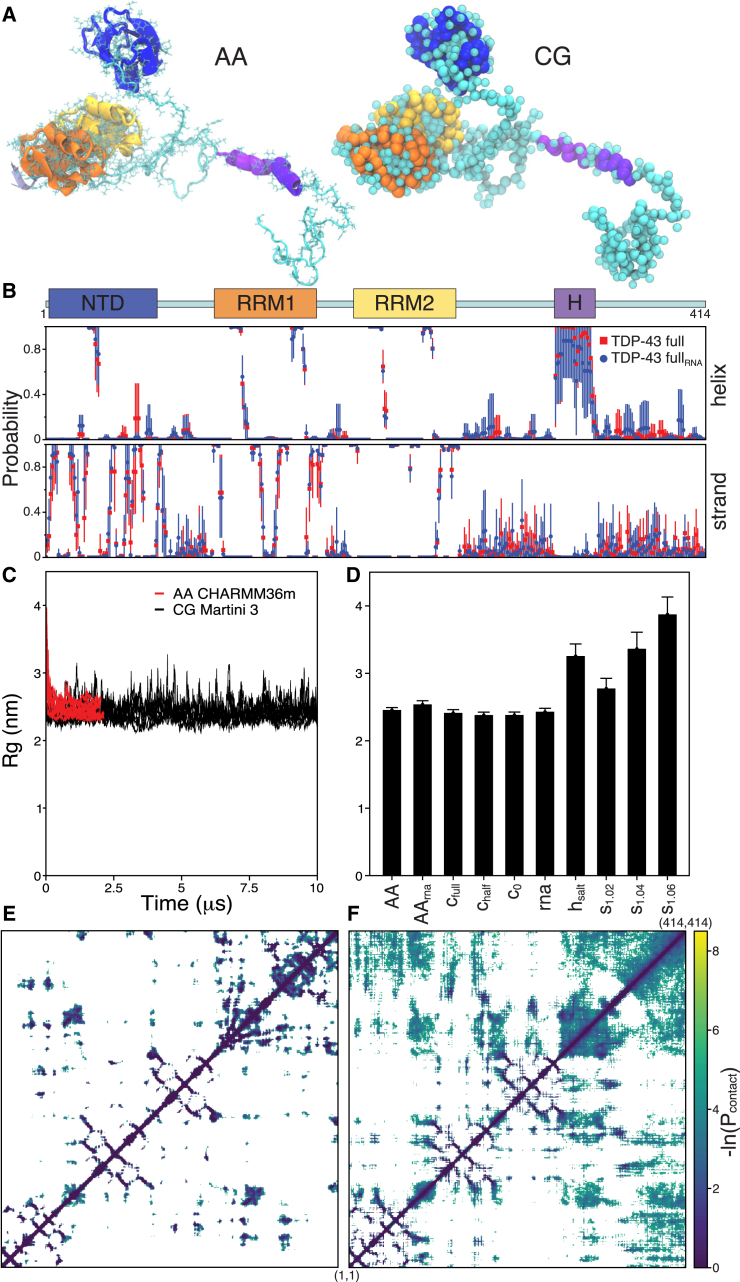
Full-length TDP-43 dynamics and self-interactions. (*A*) Models of full-length, 414 amino acid long, TDP-43 were constructed at the all-atom (AA) and coarse-grained (CG) scales. Splayed out snapshots are shown for both scales with the main structural domains color coded as shown below. (*B*) Secondary structure of AA TDP-43 shown for full-length TDP-43 with and without RNA fragment. Eight repeated simulations (from 0.2 to 2 *μ*s) are averaged and average ± SD shown (see Fig. S1 for secondary structure of additional simulations). (*C*) Radius of gyration (Rg) for TDP-43 from eight 2 *μ*s long AA simulations and eight 10 *μ*s long CG simulations. (*D*) Average TDP-43 Rg from different simulation conditions. Each is an average ± SE of eight simulations from the 0.4–2 *μ*s for the AA and 2–10 *μ*s for the CG simulations. (*E* and *F*) Residue-residue contact maps for the (*E*) AA and (*F*) CG resolutions; each is from a single representative simulation averaged over 0.2–2 *μ*s simulation time (*white* is no data and see Fig. S3 for averages of different times and repeats and Fig. S6 for averages of the different protein variant simulations). To see this figure in color, go online.

The Martini 3 (43) CG model was used to sample intra and inter protein-protein interactions of different variants of full-length TDP-43. Martini 3 is a refined version of the Martini (44) model, with a greatly increased number of CG bead types and allowing for more flexibility and finer grain tuning of molecular interactions resulting in better balanced molecular interactions (43,45). The Martinize2 program with the underlying Vermouth (46) was used to map the initial TDP-43 described above into CG coordinates and generate Martini 3 (43) topologies. Secondary structure (SS) assignment was done based on the majority structure seen in the full-length TDP-43 AA simulations (Figs. 1 and S1) except for the CTD α-helix region (for the full structure sequence, see Fig. S2 *C*). Three variants were made with different level of α-helix content in the CTD region, c_full_, c_half_, c_0_ with 27, 13, and 0 α-helix residues, respectively (Fig. S2 *D*). The Martinize2 scfix option was used, adding orientational constraints for side chains (47) and an elastic network (48) was used to maintain stable structures. Both were used only for the NTD (between residues 3 and 77), the RRM1 (between residues 105 and 177), and the RRM2 (between residues 192 and 260). Within those regions, elastic bonds were set between all backbone beads of 0.9 nm using a force constant of 500 kJ/mol/nm^2^. Additional protein variants were made from the c_full_ version: high salt (h_salt_), with scaled protein-water interactions (s_1.02_, s_1.04_, and s_1.06_), and with RNA fragment (rna) attached. h_salt_ is the same variant as c_full_ but simulated with 900 mM salt instead of 150 mM. The scaled protein-water interaction variants are also the same as c_full_ except run with the Martini bead-bead interaction matrix scaled for all protein bead interactions with water as described in (49) using scaling factors of λ = 1.02, 1.04, and 1.06. Note that increased protein-water interaction results in higher protein hydration and reduction in protein-protein interactions. For the RNA variant the 12 basepair long RNA fragment from PDB: 4BS2 (that is bound to both RRM1 and RRM2) was used. The RNA initial configurations and parameters were modeled using a preliminary nucleotide Martini 3 model. Bead mapping and bonded parameters were based on the phosphate and ribose Martini 2 (50,51). Phosphate is represented by Q5 bead while ribose by SN3a-SP1 fragment. Aromatic nucleobase rings were already available with the Martini 3. In this study the RNA fragment was used as a scaffold constraining the two RRM domains. Elastic bonds, same definition as above, were added from all RNA beads to all protein beads within RRM1 and RRM2 domains. Improper backbone dihedrals were also removed from adenine for numerical stability. Throughout the manuscript, unless otherwise specified, the unscaled full C-terminal α-helix length (c_full_) variant was used.

#### Explicit water CG simulations details

TDP-43 dynamics were evaluated using the CG Martini 3 (43) models described above. For each model three different simulation setups were run: single protein, single protein temperature scan, and two proteins. Additional systems with 24 TDP-43 proteins were set up in an elongated box with the proteins placed in a 2 × 2 × 6 configuration (spaced 8 nm apart). Eight replicas were simulated for all protein setups except the temperature scan with only one per temperature and all simulations were run for 10 *μ*s, unless otherwise specified, after initial equilibrium using GROMACS v.2021.1. All simulation times are used as is, not scaled by a factor of 4 for expected CG to AA speed conversion (44), and the aggregated total simulation time was over 2 ms. Each system was solvated, charge neutralized and 150 mM NaCl added (except for the high-salt system that had 900 mM NaCl) using the system builder *insane* (52). The resulting number of CG water beads (each representing four water molecules) was ∼23k, ∼33k, and ∼93k for the 1, 2, and 24 protein systems respectively. For systems with more than one protein, initial configurations for each protein in each repeat were randomly sampled from a library of initial configurations. An initial configuration library was constructed for each protein model variant from an equivalent single-protein simulation; over the 10 *μ*s simulation snapshots were saved every 10 ns for a total of 1000 possible structures. The simulations were started with 1500 steps of energy minimization followed by three initial equilibrium steps using 1, 5, and 20 fs time steps totaling a little over 2 ns. The initial steps used the Berendsen thermostat and barostat (53), while production runs used the velocity rescaling thermostat (54) and the Parrinello-Rahman barostat (55) coupled with a temperature of 310 K and 1 bar isotropic pressure. The simulations were run with the new-rf Martini parameter set (56).

#### Implicit water, one-bead-per-residue CG simulations

Coexistence slab simulations of TDP-43 that use a single bead per amino acid resolution were conducted using the HOOMD-Blue 2.9.7 software package (57), following the protocol described previously (58,59). The folded domains, namely NTD, RRM1, and RRM2, and the conserved helix region (amino acids [aa] 320–343) of CTD were fixed as in the initial full-length TDP-43 structure using the rigid-body constraint (60). We employed the hoomd.md.constrain.rigid command in HOOMD to restrain the C_ɑ_ atoms and maintain the structure of folded domains. The initial slab configuration (20 × 20 × 168 nm) was prepared from the 100 TDP-43 proteins using a coarser implicit solvent model, “HPS-Urry” (61), as described in (62). This model has been recently validated for accurately describing the phase behavior of a multidomain protein with folded regions, namely HP1α (63). For the production simulations, we conducted a 3.5 *μ*s NVT ensemble simulation at a temperature of 310 K using the Langevin thermostat with a residue friction factor, γ = m_AA_/τ. Here, m_AA_ is the mass of each amino acid bead and τ is the damping parameter, which was set to 1000 ps. The time step was set to 10 fs. In addition to the coexistence slab simulations, we also performed single-chain simulations of TDP-43 WT and a six Trp to Ala mutations variant (6WtoA) at 310 K using the LAMMPS software package (64). In these simulations, the folded domains (NTD, RRM1, and RRM2) and the CR (aa 320–343) were kept rigid, using the “fix rigid” command. We utilized in-house scripts for calculating the radius of gyration (Rg), density profile, and contact map, as described in reference (59).

#### Simulation analysis

The AA and explicit water CG simulations were analyzed using a combination of GROMACS tools and custom analysis written in python using MDAnalysis (65,66). For contact analysis, any residues with beads within 0.8 nm are counted as in contact, unless otherwise specified, and the average residue-residue contacts are the time-averaged number of residues in contact with each residue over the analyzed part of the simulation. SS was assessed using the DSSP algorithm (67,68) and all snapshots were made with VMD (69).

Most of the simulation inputs and parameter files as well as examples of simulation snapshots will be made available at Biochemical and Biophysical Systems Group.

## Results and discussion

### TDP-43 structure and modeling

To build a full-length TDP-43 structure several available TDP-43 fragments as well as constructed intrinsically disordered regions (IDRs) were evaluated (Fig. S1). A full-length structure was derived by merging different stable structural fragments and generating the IDRs in between (Fig. 1 *A*). AA simulations of the full-length protein structure, both with and without an RNA fragment bridging the two RRM domains, were simulated. Fig. 1 *A* shows the average SS assignment from eight 2 *μ*s long AA simulations of both variants. Additional SS elements as well as analysis of fragment simulations are shown in Fig. S1. The simulations all show stable structures of the NTD and two RRM domains with only small SS elements in the linker/IDR. However, the C-terminal helix was metastable having some simulations and/or simulation parts with full-helix and half-helix, as well as instances of mostly unstructured (no-helix). TDP-43 C-terminal looks to be able to adopt different levels of helix content, and helix prevalence is likely dependent on protein environment. Comparison of the full-length structure constructed here to a subsequent prediction of TDP-43 structure by AlphaFold (70) shows significant similarity in the SS (Fig. S2). The main difference is the length of the C-terminal helix with the AlphaFold prediction helix length close to the half-helix.

The full-length TDP-43 structure, and structural stability as seen in the AA simulations, was used to create several CG Martini 3 model variants where a full-length TDP-43 was explored with and without an RNA fragment connecting the RRM domains. Due to the metastable structural variation seen in the C-terminal helix region in the AA simulations, three CG models were constructed with full, half, and no C-terminal α-helix content (Fig. S2 *D*).

### TDP-43 self-interactions

At both the AA and CG scale, full-length TDP-43 collapses readily to a quite compact form (within 100 ns). In its compact form TDP-43 has numerous self-residue interactions, especially between the disordered CTD and the rest of the protein. Those interactions, however, are dynamic allowing the protein to readily rearrange itself. Fig. 1 *C* shows the variation of the Rg with time for the eight repeated AA full-length simulations as well as the eight full-length CG simulations with full C-terminal helix (c_full_). The AA and CG models exhibit comparable Rg, with a somewhat higher dynamic range in the CG simulations. The average Rg was also evaluated for all the different full-length variants tested, i.e., with half or no helical content of the CTD (Fig. 1 *D*), with no significant differences observed. Fig. 1, *E* and *F* show residue-residue contact maps from representative AA and CG simulations, respectively. The overall contacts are similar, showing contacts within the proteins SS domains, some contacts between the domains, and significant contacts within the disordered CTD and between the CTD and other protein parts. The contacts in the CG simulation are more disperse due to the faster sampling and smoother interaction potentials (31,44). Residue-residue contact maps averaged over all eight repeats are shown in Fig. S3, demonstrating the relative affinity for different contact sites.

Full-length TDP-43 simulated with the AA CHARMM36m or CG Martini 3 models behaved overall similarly. In both models the protein is rather compact with an Rg of 2.46 ± 0.04 and 2.41 ± 0.05 nm (average ± SE) for AA and CG c_full_, respectively. In a recent benchmark of AA force fields for the IDP FUS, a significant difference in Rg was observed. Of the nine force fields tested CHARMM36m was the second most compact (71). Protein-protein interactions have been shown to be more accurately represented in Martini 3 compared with Martini 2 (43,45) and the salt-dependent coacervation of Martini 3 poly-lysine and poly-glutamate systems captured correctly (72). For IDPs, however, applying a protein-dependent scaling factor for the interaction potentials resulted in better agreement to Rg from SAXS (49,73,74). To the best of our knowledge the Rg for WT full-length TDP-43 has not yet been determined, but Wright et al. (28) purified a TDP-43 mutant with increased solubility, where all six tryptophans had been converted to alanine. This mutant had a Rg of ∼4.1 nm which should be an upper limit for WT TDP-43. To evaluate more extended TDP-43 configuration and to test the CG model sensitivity, we explored scaled protein-water interactions (49,73) (λ = 1.02, 1.04, and 1.06), and conditions with high salt (h_salt_, 900 instead of 150 mM NaCl) and simulations at higher temperature (Fig. S4). The different parameters and conditions resulted in a higher average Rg and a more frequent sampling of extended structures (Fig. 1 *D*). In addition, CG Martini 3 simulations of the WT c_full_ modified to include the six Trp to Ala mutations variant (6WtoA) as in Wright et al. (28) resulted in slightly higher Rg (Fig. S5), by about 4%. These tryptophan to alanine mutations are made from initial c_full_ coordinates and have the same SS assignment and as such are not expected to capture the full effect of the mutations.

### TDP-43 cross-interactions

**Figure 2:**
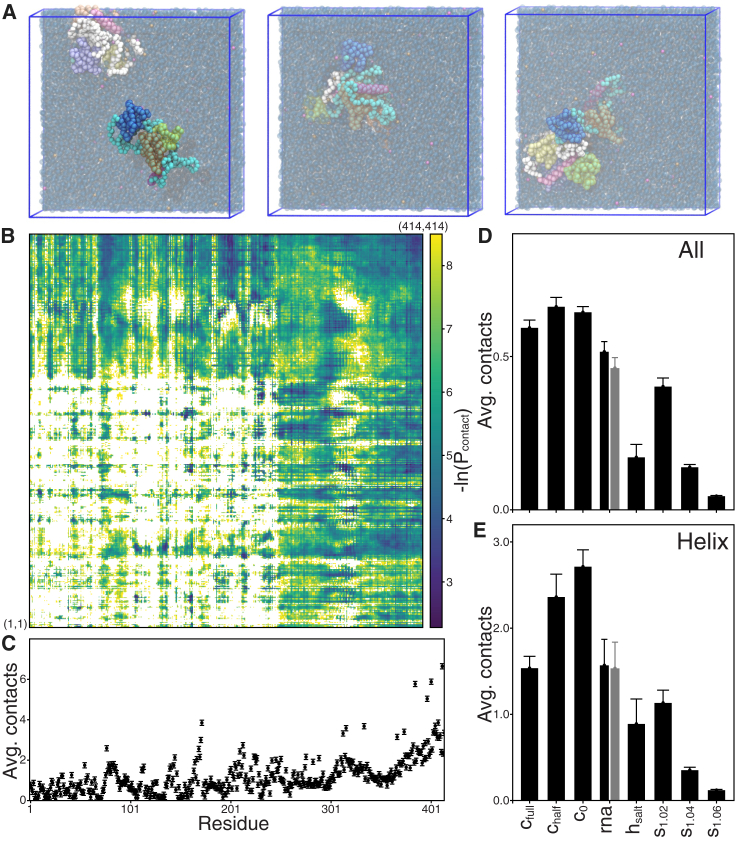
TDP-43 cross-interactions. (*A*) Representative snapshots from one c_full_ simulation, the backbone of the two TDP-43 proteins is colored differently in cyan and white, and the structure domains are colored according to Fig. 1*A* with the backbone beads of the two proteins in cyan and white. The left snapshot is from early in the simulation before the two proteins associated. The middle and right snapshots are from close to the end of the simulations and 15 ns apart. (*B*) Residue-residue contact map between the two c_full_ proteins, averaged over the eight simulation repeats excluding the first 2 *μ*s of each simulation. (*C*) Average residue-residue cross-interaction contacts shown for each protein residue, error bars are SE between the eight simulation repeats. (*D*) Total average contacts between the different protein variants tested and (*E*) the average contact for the helix region (residues 311–360) only (error bars are SE between the eight simulation repeats). For systems with bound RNA, average contacts are shown including (*black*) and excluding (*gray*) RNA. Contact maps and average contacts per residues for the all the variants are shown in Fig. S7. To see this figure in color, go online.

Utilizing the computationally efficient CG Martini model makes it possible to sample the systems more effectively, simulate larger systems for longer times and to explore more model variations. We studied TDP-43 protein-protein cross-interactions by simulating two full-length TDP-43 proteins for all modeled protein variants (Fig. 2 *A*). Two proteins, randomly sampled from a library of initial configurations, were placed apart and simulated for 10 *μ*s with eight replicas (using different initial configuration) for each protein variant. At the start of the simulations the two proteins diffused individually until they happen to come into contact, which for the unscaled and regular salt conditions normally leads to strong but dynamic protein-protein association. The number of contacts between the two proteins fluctuates significantly over time and their relative orientation/configurations change continuously (e.g., Fig. 2 *A*). However, no dissociation of the dimers is observed at the 10 *μ*s timescale for the unscaled and regular salt conditions. To evaluate the TDP-43 cross-interactions, we defined each residue to be in contact if any CG residue bead was within 0.8 nm of another. For the c_full_ protein variant the interprotein residue-residue contact map (Fig. 2 *B*) and the average contact per residue (Fig. 2 *C*) are given. Unlike the intraprotein contact map (Figs. S3 and 1 *F*) the residue contacts within the stable structural domains are not present between proteins, instead interprotein contacts are reduced between the domains mostly due to buried inaccessible residues. Like the intraprotein contact, the disordered CTD forms the greatest number of contacts both to itself and to other protein domains (Fig. 2, *B* and *C*). The same analysis for the other simulated protein variants is shown in Fig. S7, the number of contacts per residue averaged over the full protein is shown in Fig. 2 *D*, and the average only over the C-terminal helix region (residues 311–360) in Fig. 2 *E*. The different protein variants overall form similar types of contacts as c_full_ but with some notable exceptions. When the length of the C-terminal helix is reduced (c_full_ to c_half_ to c_0_) the number of contacts within the helix region goes up, both for the intra- (Fig. S6) and interprotein contacts (Fig. S7). The total average contacts for c_full_, c_half_, and c_0_ are about the same (Fig. 2 *D*), whereas the contacts within the helix region go up sharply with reduced helix content (Fig. 2 *E*). Including an RNA fragment spanning both RRM domains greatly reduces the conformational flexibility of those domains substantially decreasing intradomain contacts (Fig. S6) as well as interdomain contacts (Fig. S7), but the overall number of contacts is only modestly decreased whether counting with or without RNA (Fig. 2 *D*). This agrees with the increased solubility of TDP-43 in the presence of UG-rich RNAs (22) that bind to both the tandem RRMs. Increasing the salt concentration (h_salt_) results in an overall reduced protein-protein interaction and number of interprotein contacts (Figs. 2 *D* and S7), consistent with the inhibitory effect of salt on TDP-43 phase separation (15).

In these two TDP-43 protein simulations, few NTD-NTD contacts and no full NTD dimerization are observed. The lack of observed dimerization is presumably due to lack of sampling (see assemblies simulations below) and/or needed changes in SS, the currently tested Martini models based their NTD domain on the monomeric PDB: 5MRG structure, which differs somewhat from the dimeric structure in PDB: 6B1G (Fig. S1). As expected, the CR-CR contacts are among some of the most prominent contacts observed (Fig. S7) but the number of contacts goes up with decreased helix length; c_full,_ c_half_ to c_0_ (Fig. 2 *E*) is contrary to previous experimental results (33,35). Increasing the protein-water interaction by scaling the Martini 3 bead-bead interactions (λ > 1) results in an overall reduced protein-protein interaction and number of interprotein contacts (Figs. 2 *D* and S7), with a modest decrease for s_1.02_, significant reduction for s_1.04_, and very few contacts for s_1.06_. The scaled simulations result in much more uniform sampling of the CR-CR contacts (Fig. S7), which is also observed in the assemblies simulations (see below), indicating that the c_half_ and c_0_ simulations, with their increased conformational flexibility, are compensating for a likely lack of sampling and/or imbalance of the interaction strength between structured and disordered domains in the c_full_ unscaled simulations. Suggesting that the unscaled simulations are less reliable and the scaled Martini 3 simulations better capture TDP-43 dynamics.

### TDP-43 assemblies

**Figure 3:**
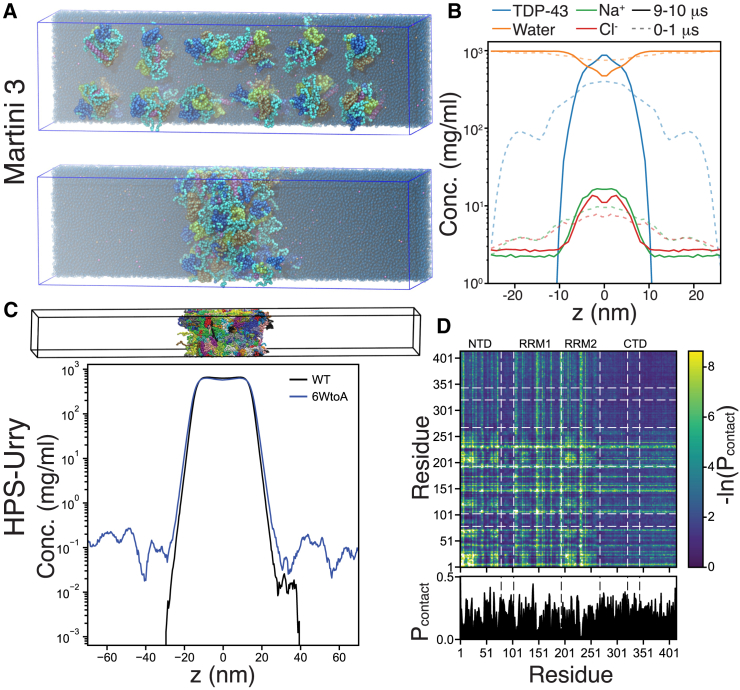
TDP-43 assemblies. (*A*) Representative snapshots from CG Martini 3 simulations capturing TDP-43 assemblies, shown for a simulation with 24 TDP-43 c_full_ at time 0 and after 10 *μ*s of simulation where all TDP-43 proteins have assembled. Proteins are colored according to the same scheme as in Fig. 2*A*, with all protein back bone beads in cyan. (*B*) Relative distributions of CG Martini 3 particles along the z-dimension of the slab, relative to the center of the box and averaged over the first (*dotted light lines*) and the last (9–10 *μ*s, *solid lines*) microsecond of the simulation. (*C*) Representative snapshot of TDP-43 condensate from a CG slab simulation based on the HPS-Urry model (*top*). The concentration profile (*bottom*) of TDP-43 WT versus 6WtoA along the z-dimension of the slab at 310 K. (*D*) Pairwise intermolecular contact map (*top*) and per-residue contact probabilities (*bottom*) in the condensed phase from the WT HPS-Urry CG simulations. To see this figure in color, go online.

Using the CG Martini resolution, it is possible to simulate multiple TDP-43 proteins and their assemblies into mini granules, but mounting computational cost limits the number of proteins that can used in these studies. Here, we illustrated the possibility of using Martini 3 resolution modeling by simulating the assembly of 24 full-length TDP-43 molecules (Fig. 3 *A*). Eight simulations with 24 proteins spaced out in an elongated water box were started and each simulated for over 10 *μ*s (Video S1 shows the full assembly process for one of the simulations). Five of the eight simulations end in a single assembly of all 24 proteins. The remaining three simulations include two protein clusters that span the periodic boundary condition preventing them from diffusing and merging. In all cases, individual TDP-43 molecules associate quickly and cluster within the first few hundred ns. A handful of assemblies break and reform, but no individual TDP-43 proteins disassociate after they have properly integrated at the simulation timescales assessed. The resulting TDP-43 assemblies are too small to form a bulk condensed phase, but appear to be approaching bulk phase properties. The assemblies are fluid like, each TDP-43 molecule has numerous contacts with other molecules, but those contacts are dynamic and the molecules are quite mobile within the assembly (Video S1). Significant water is still present within the assemblies with minimum water density a little less than half that of bulk (Fig. 3 *B*), ions are significantly enriched compared with bulk (Fig. 3 *B*), and both water and ions exchange readily with the bulk phase (75). Extending the simulations for two assemblies for a further 5 *μ*s at both unscaled (c_full_) and the different scaled (s_1.02_, s_1.04_, s_1.06_) protein-water interactions resulted in stable assemblies in all cases except for s_1.06_, which started to break apart. Unscaled and s_1.02_ showed similar protein density in the assembly while s_1.04_ and s_1.06_ showed larger assemblies with reduced protein density (Fig. S8 *A*). The average Rg for individual proteins within the assemblies show a corresponding increase with scaling and notably in all cases Rg is larger in the assemblies then in simulations of single proteins in solution (Fig. S8 *B*). Protein diffusion within the assemblies also reveals their fluid-like nature: the protein diffusion goes up sharply with increased protein-water interactions (Fig. S8 *C*). Pairwise intermolecular contact maps (Fig. S9) showed increased sampling compared with the two protein simulations (Fig. 2) with less C-terminal helix exclusion with the full helix length model and more interactions in the NTD-NTD region. From the assemblies simulations and the Rg comparison, the above scaling of the protein-water interactions is likely to capture more accurate TDP-43 behavior at the Martini 3 level, and appropriate λ scaling is likely to be somewhere between 1.02 and 1.05. Note, simulation with inappropriately higher or low scaling can still be of great value and effectively capture relative interaction and interaction mechanisms, but absolute energetics and dynamics would be less trustworthy.

Video S1. Process of TDP-43 assemblies The video shows a Martini 3 simulation of 24 full-length TDP-43 proteins. For each protein the backbone beads are shown in cyan and the N-terminal domain (NTD), the two RNA recognition motifs (RRM1 and RRM2), and C-terminal α-helix are colored in blue, orange, yellow, and purple, respectively. The borders of the simulation box are shown in blue, protein backbone beads in periotic images above and below the box are shown in gray, and three-quarters of the water and ions removed for clarity. Initially the 24 proteins are placed apart, but they quickly start associating, forming larger assemblies, which coalesce into a single assembly. The proteins interact strongly but remain dynamic and solved with significant amount of water and ions (Fig. 3, *A* and *B* in main text).

To study larger-scale assemblies of multidomain proteins and effectively explore their behavior under different conditions, one can use a coarser and more computationally efficient representation of amino acids as single beads and the speed of the conformational sampling can be further increased by representing the solvent in an implicit manner (58). Here, we used a sequence-specific, implicit solvent, model based on the Urry hydrophobicity scale (61) to simulate the monomers of both TDP-43 WT and 6WtoA variants. From the computed Rg distributions of these two variants (Fig. S10), we observe that the 6WtoA ensemble (mean Rg = 3.507 ± 0.024 nm) is more expanded compared with WT (mean Rg = 3.176 ± 0.035 nm), but still less extended than the Rg values obtained from SAXS experiments (28). This discrepancy could be related to the potential unfolding of folded domains in the experiments since 3 out of 6 tryptophans in TDP-43 WT are located within folded domains: 68W is buried within NTD (PDB: 5MRG), while 113W and 172W are exposed on the surface of RRM1 (PDB: 4BS2). The use of restraints for folded domains does not permit us to capture the effect of potential destabilizing mutations on folded domains and likely accounts for the discrepancy in Rg between our CG HPS-Urry or the Martini simulations and SAXS experiments. Subsequently, we simulated the condensed phase of TDP-43 WT and 6WtoA variants and analyzed the underlying interactions that stabilize it. The slab geometry (58) that is utilized allows efficient sampling of the coexisting phases at a reasonable computational cost (Fig. 3 *C*, *top*). Fig. 3 *C* shows the equilibrium concentrations of TDP-43 WT and 6WtoA in dense and dilute phases as a function of the z-coordinate along the slab configuration. The 6WtoA variant shows a significant destabilization of the condensed phase compared with TDP-43 WT, as demonstrated by an increase in the saturation concentration (c_sat_) compared with WT estimated from the density profiles (Fig. 3 *C*). The analysis of pairwise, intermolecular contact maps of TDP-43 WT (Fig. 3 *D*) indicates that a multitude of interdomain interactions collectively stabilize the condensed phase. Similar to the CG Martini results, CTD shows both homotypic (CR-CR and IDR-IDR) and heterotypic interdomain contacts. As can be seen from per-residue contact probabilities (Fig. 3 *D*), CTD-CTD interactions involve both conserved helix and disordered flanking regions (76). The two protein CG Martini simulations did not show very prominent NTD-NTD contacts (Figs. 2 *B* and S7), but the Martini assemblies simulations show more pronounced NTD-NTD contacts (Fig. S9), and the coarser HPS-Urry CG model readily shows NTD-NTD interactions from different patches of NTD (Fig. S11). Specifically, it highlights favorable interactions between the oppositely charged segments (aa 8–23 and 48–54), which is consistent with the head-to-tail dimerization proposed in the literature (15,29). Interestingly, RRMs interact with both NTD and CTD, which hints at their additional roles in the LLPS of TDP-43. Overall, residue-level CG phase coexistence simulations suggest the cooperativity between individual domains for condensed phase stability, in addition to the NTD/CTD oligomerization.

## Conclusions

To effectively capture TDP-43 dynamics, multiscale simulations are needed, with models capable of resolving detailed protein residue interaction, protein-protein interaction, and all the way to LLPS and cellular scale TDP-43 filamentation. Here, we present a full-length TDP-43 structure model that we explored at AA, explicit, and implicit water CG resolutions. At their intersections each model exhibits comparable overall properties and utility in resolving TDP-43 molecular-level interactions at their scale. The different models, however, should be used with care, respecting their limitations, and often multiple models need to be evaluated, e.g., when SS and/or stable tertiary structure changes different CG models might need to be constructed. We hope these models will help with further studies of molecular-level interactions of TDP-43, TDP-43 variants, and interaction with other molecules as well as provide a foundation for building models resolving TDP-43 dynamics at larger scale.

TDP-43 has a strong self-affinity, with most protein configurations being rather compact and TDP-43 proteins clustering readily into protein assemblies. For both inter- and intraprotein-protein interactions there are numerous common interactions that are spread over most of the protein, but the highest number of contacts are within the disordered CTD, particularly in the CTD’s CR-CR and IDR-IDR, as well as between the CTD and the rest of the protein. The large number of favorable protein contacts leads to their high self-affinity with a myriad of different interaction modes, allowing TDP-43 proteins to readily self-assemble but still exhibit fluid properties, i.e., within TDP-43 assemblies, protein exchange position and water and ions diffuse throughout.

**Figure 4:**
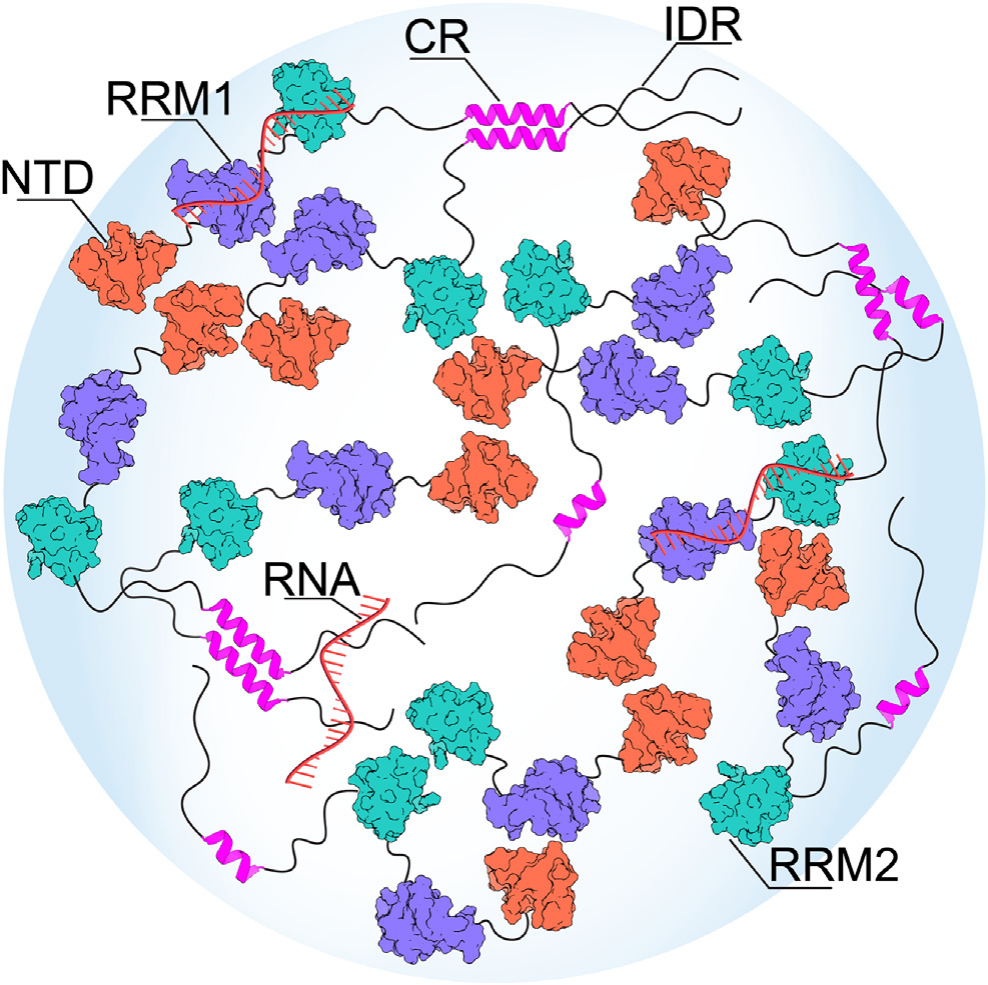
A diverse array of interdomain interactions collectively stabilize the TDP-43 condensed phase. The balance between NTD-NTD (via head-to-tail) and CTD-CTD (facilitated by CR-CR and impacted by IDRs) interactions critically determines the formation of soluble, liquid-like condensates. UG-rich RNAs play an important role in maintaining the solubility of TDP-43 by binding to tandem RRMs and CTD (via RGG motifs). Simulations performed in this study suggest that RRMs can also interact with both NTD and CTD, which hints at their additional roles in modulating the LLPS of TDP-43. To see this figure in color, go online.

Multiscale simulations in combination with existing literature and/or new experiments could help us to elucidate the mechanistic aspects of TDP-43 phase behavior. By integrating key intermolecular interactions observed in our simulations with findings from the literature, we propose a mechanistic picture of TDP-43 condensate formation, which is governed by a diverse set of interactions at the domain level (Fig. 4). Homooligomerization by NTD (head-to-tail arrangement) (15,16) and CTD (via both conserved helical and disordered flanking regions) (33,35,76) domains greatly enhance the propensity of TDP-43 to form liquid-like condensates, which maintain the solubility in the presence of UG-rich RNAs that bind to both the tandem RRMs (22) and CTD, likely via RGG motifs (33). Other interdomain interactions that remain elusive so far may play additional roles in modulating the LLPS of TDP-43. These findings shed light on the underlying mechanisms driving TDP-43 condensate formation and provide valuable insights into its functional regulation.

## Author contributions

H.I.I., X.L., A.R., and P.M. set up, ran, and analyzed the AA, CG, and implicit solvent CG simulations, P.C.T.S. and S.J.M. developed the Martini 3 RNA prototype model. All authors contributed to the design of the work and wrote the paper.

## Supporting material

Document S1. Figures S1–S11

Document S2. Article plus supporting material
