# Supplementary material for: Multiscale simulations reveal TDP-43 molecular-level interactions driving condensation
Source: Biophys J. 2023 Oct 17;122(22):4370–81. doi: 10.1016/j.bpj.2023.10.016 (PMC10720261; doi:10.1016/j.bpj.2023.10.016)
Supplement: Document S1. Figures S1–S11 [file mmc1.pdf]

Biophysical Journal, Volume *122*

Supplemental information

Multiscale simulations reveal TDP-43 molecular-level interactions driving condensation

Helgi I. Ingólfsson, Azamat Rizuan, Xikun Liu, Priyesh Mohanty, Paulo C.T. Souza, Siewert J. Marrink, Michael T. Bowers, Jeetain Mittal, and Joel Berry

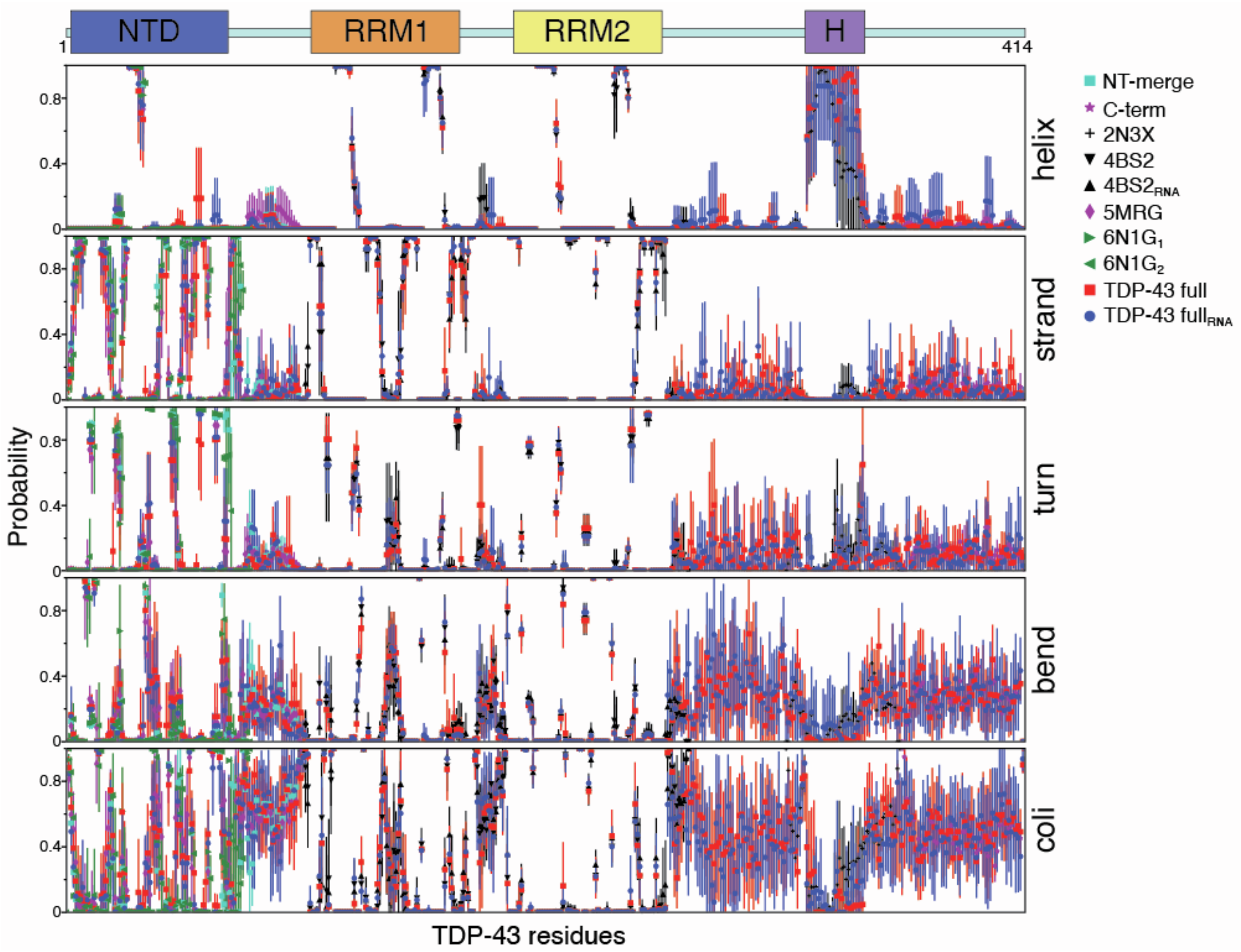

FIGURE S1 Secondary structure of TDP-43 determined from AA CHARMM36m simulations. Results from both full-length TDP-43 simulations and simulations of all structural fragments are combined to show protein regions with conserved and variable secondary structure assignment. Simulations were run for full-length TDP-43 both with and without RNA fragment and different TDP-43 fragments: for the NTD using pdb 5MRG and 6N1G (dimer structure each monomer is shown separately as $6N1G_1$ and $6N1G_2$), as well as NT-merge which is an extension of 6N1G chain B using 5MRG; for RRM1 and RRM1 pdb 4BS2 was used with and without RNA fragment; and for the C-term the helix pdb 2N3X was simulated and the remaining C-term residues (361-414 modeled as colie in MOE). For each condition eight simulations were run for 2 µs here showing average +/- SD between those over the last 1.8 µs.

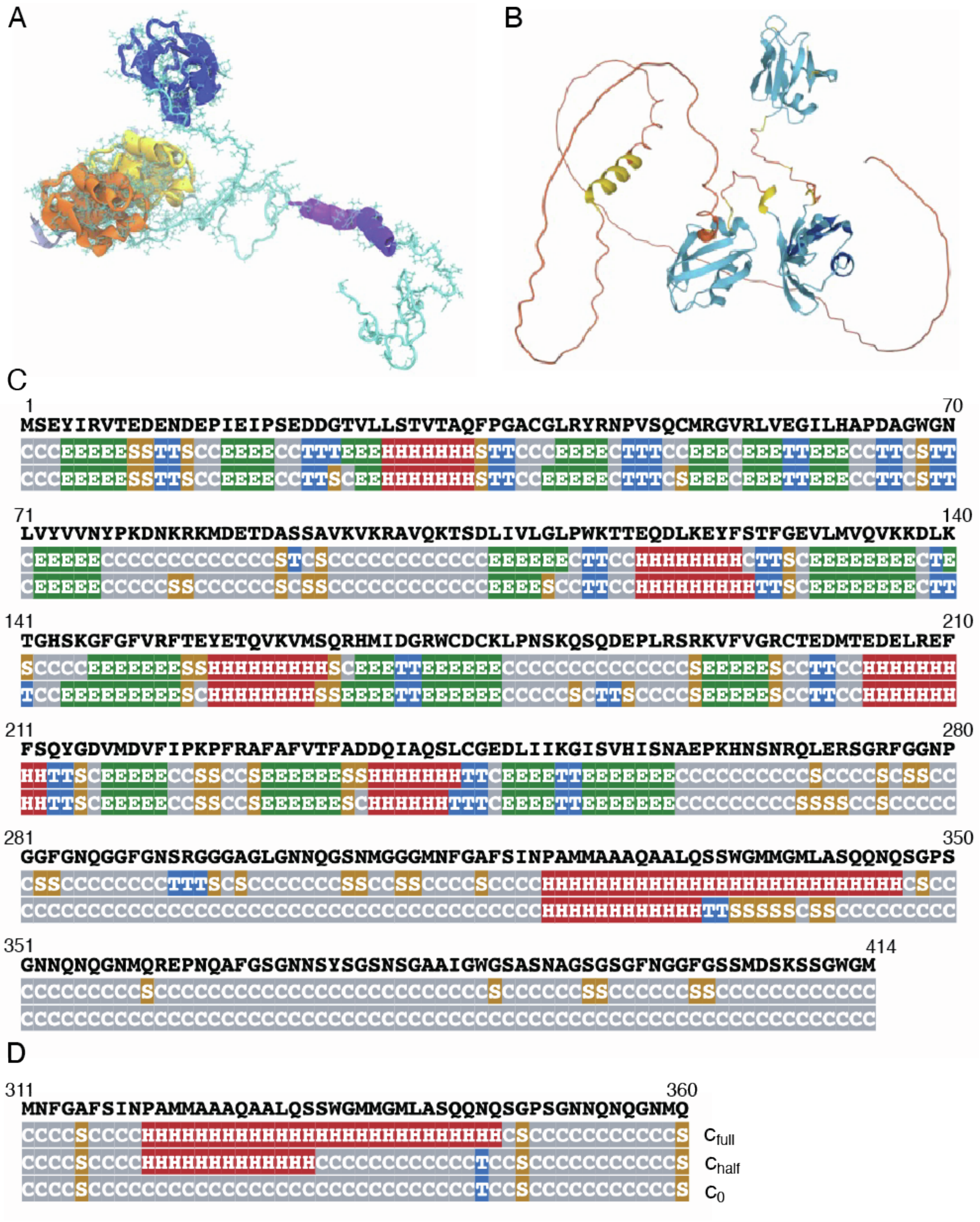

FIGURE S2 Full-length models of TDP-43. (A) In this work a full-length model of TDP-43 was generated by combining available structures, evaluating secondary structure stability using in AA simulations and generating unstructured regions using MOE. Same image of c_{full} is shown in Fig. 1A of

the main text with explanation of coloring. (B) Subsequent Alphafold prediction shows an overall similar structure. Image is from www.uniprot.org/uniprotkb/Q13148 and shows AlphaFold (1) prediction identifier AF-Q13148-F1. The colors indicate per-residue AlphaFold confidence score (pLDDT), with blue, light blue, yellow, and orange representing pLDDT of >90, 90-70, 70-50, and >50, respectively. (C) Secondary structure analysis of both structures (A, top, and B, bottom) are very similar except the AlphaFold prediction C-term α-helix is shorter very close the helix length in c_{half}. (D) Secondary structure of the (c_0., c_{half}., c_{full}) C-term α-helix variants are shown. The C-terminal α-helix, initially modeled from pdb 2N3X (residues 311-360) is metastable in the AA simulations (Fig. 1C and S1) and therefore modeled in three possible secondary structure configurations with helix length 0, half and full.

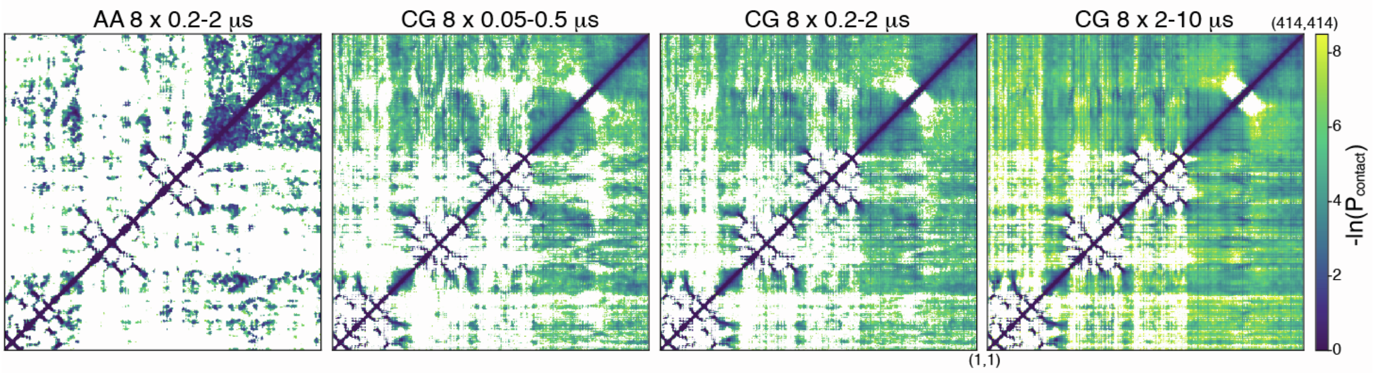

FIGURE S3 Full-length TDP-43 residue-residue contact maps for the AA and CG resolutions. Each matrix shows the average contact fraction of eight repeated full-length TDP-43 simulations without RNA and using the c_{full} model in the CG simulations. For each repeat residue-residue contacts are calculated over 0.2-2 μs of the simulations for AA and for the CG three different window sizes are shown 50-500 ns, 0.2-2 μs and 2-10 μs. Note, due to the smoother interaction potentials in the CG force field the dynamics are normally faster, often estimated about 4-fold faster (2,3).

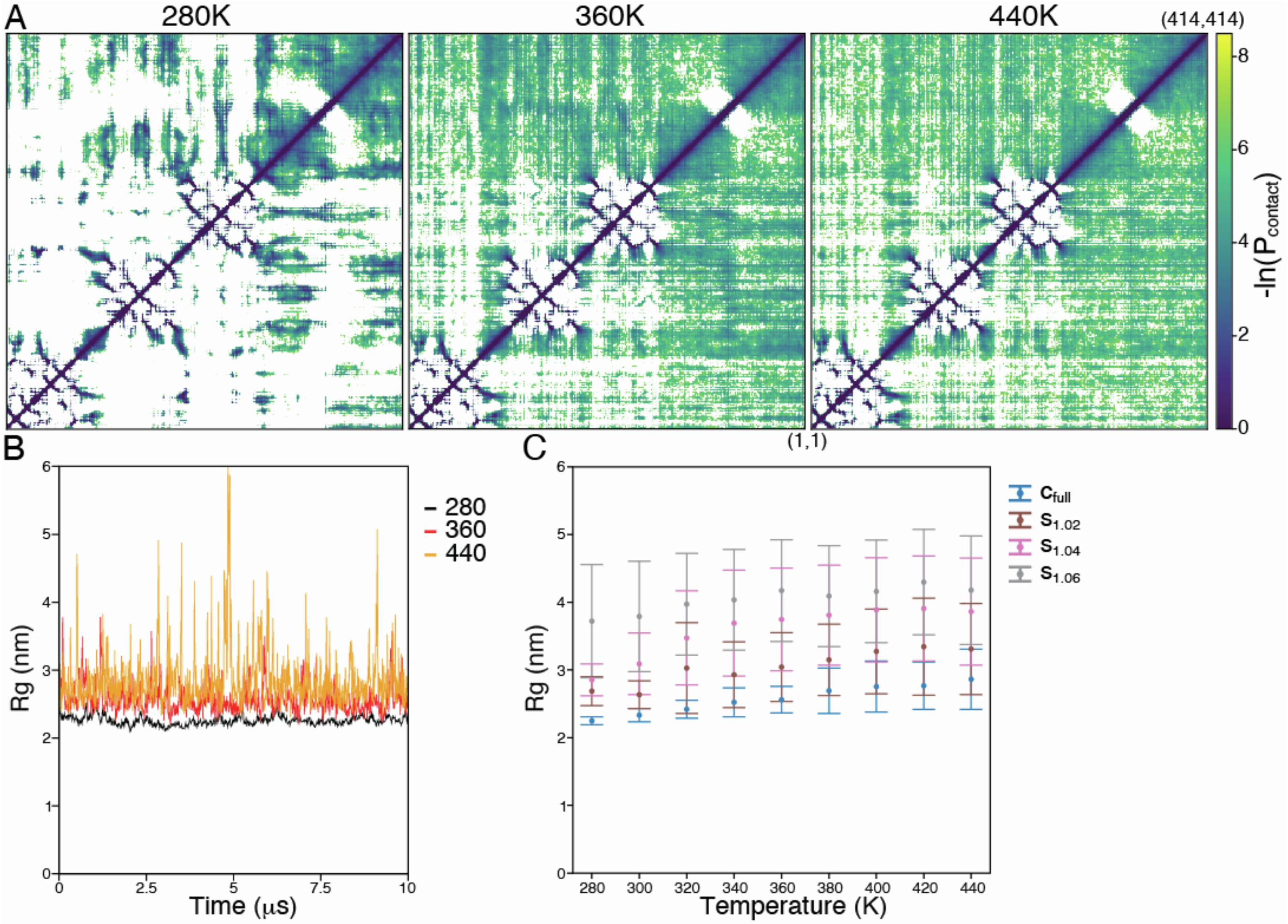

FIGURE S4 TDP-43 self-interactions at different temperatures. Self-interactions of full-length TDP-43 were evaluated in CG simulations ranging from 280K to 440K in 20K intervals. Each simulation was run for 10 μs. (A) Representative residue-residue contact maps showing the probability of contact for c_{full} at 280K, 360K and 440K from one simulation at each temperature and averaged over 2-10 μs. (B) Radius of gyration (Rg) with time for the same three simulations as shown in A. (C) Average TDP-43 Rg at different temperatures for the c_{full} model using the regular Martini 3 protein water interactions (c_{full}) and scaled by 1.02, 1.04 and 1.06 ($s_{1.02}$, $s_{1.04}$, and $s_{1.06}$). Avg±SD over 2-10 μs of each simulation are shown.

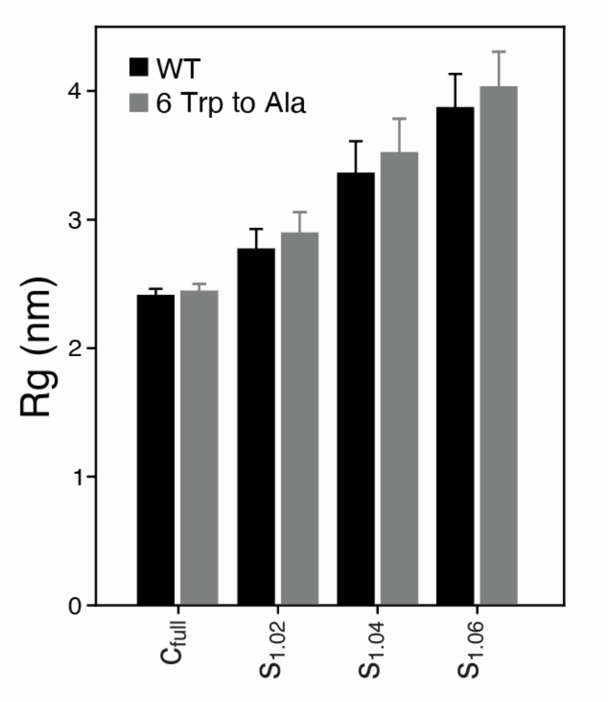

FIGURE S5 TDP-43 radius of gyration (Rg) comparing WT to the six Trp to Ala mutant (6WtoA) used in Wright et al. (4). Average Rg are shown for the c_{full} model using the regular Martini 3 protein water interactions (c_{full}) and scaled by 1.02, 1.04 and 1.06 ($s_{1.02}$, $s_{1.04}$, and $s_{1.06}$). The WT results shown with black bars are the same results shown in Fig. 1D and the six Trp to Ala mutant results are shown with gray bars. Note, the Trp to Ala mutations are made from initial c_{full} coordinates and same secondary structure assignment as WT c_{full} and therefore not expected to capture the full effect of the mutations. Each is an Avg±SE of eight simulations from the 2-10 μs for the CG simulations.

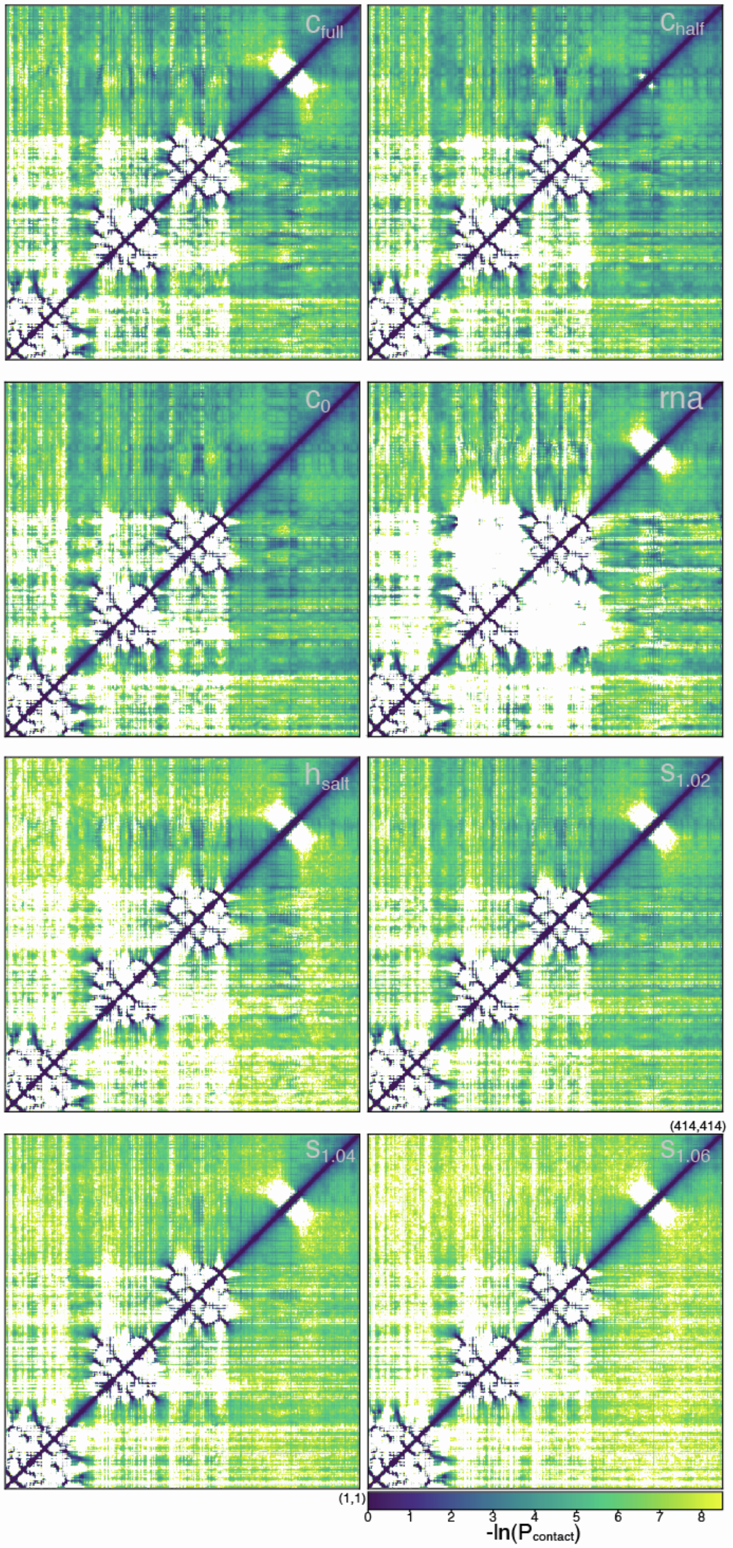

FIGURE S6 TDP-43 self-interactions for all protein variants. Residue-residue contact maps between residues in the full-length TDP-43 protein. For each protein variant the contacts are averaged over the eight simulation repeats excluding the first 2 μs of each simulation. Same as Fig. 1E CG but here shown for all protein variants tested.

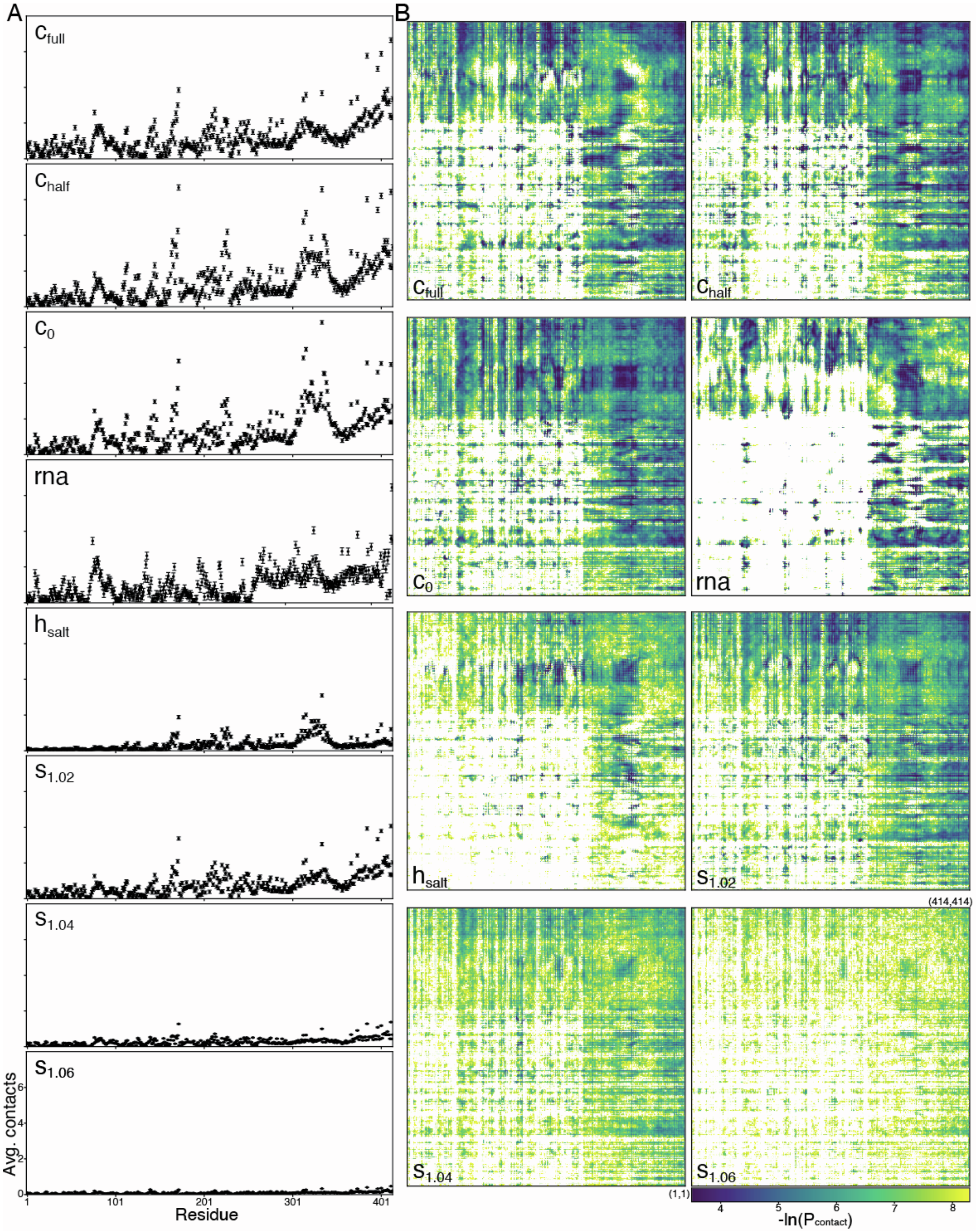

FIGURE S7 TDP-43 cross-interactions for all protein variants. (A) Average residue-residue cross-interactions contacts (Avg±SE between the eight simulation repeats) and (B) residue-residue contact maps between the two proteins. For each protein variant the contacts are averaged over the eight simulation repeats excluding the first 2 μs of each simulation. Same as Fig. 2B and C but here shown for all protein variants tested.

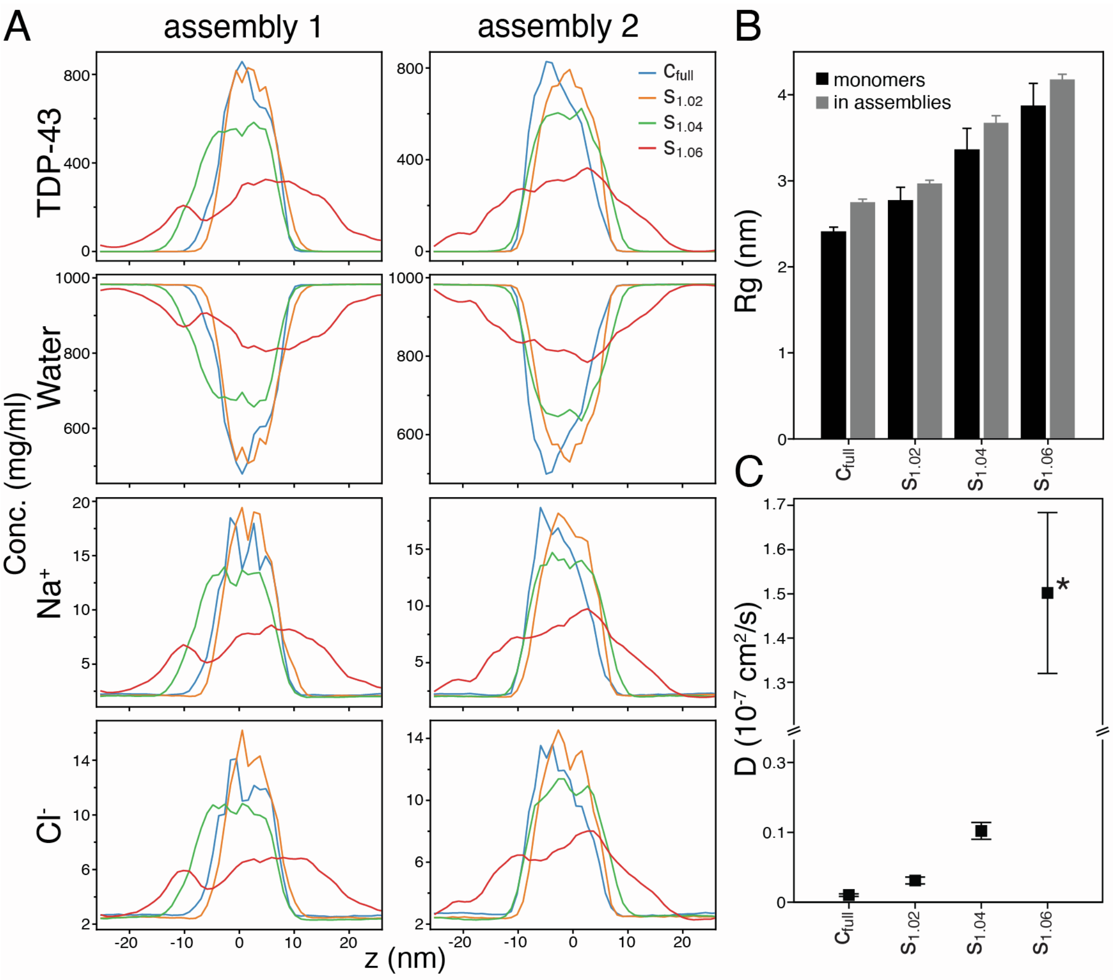

FIGURE S8 TDP-43 assemblies with scaled protein-water interactions. Two well-formed assemblies were selected from the eight c_{full} 24 protein unscaled simulation sets (see Fig. 3A and B). The simulations were continued past the original 10 μs for another 5 μs, using protein-water λ scaling of 1, 1.02, 1.04, and 1.06 (c_{full}, $s_{1.02}$, $s_{1.04}$, and $s_{1.06}$, respectively), and the last 4 μs used for analysis. (A) Relative distributions of CG particles along z-dimension of the slab, relative to the center of the box, shown for the two different initial configuration assemblies. (B) Rg for the TDP-43 molecule as single protein (monomers) in solution (black bars, Avg±SE between the eight repeated simulations) and averaged over each TDP-43 protein in the assemblies (gray bars, Avg±SE between all 48 proteins, 24 in each of the two assemblies). (C) Protein diffusion was determined for each protein in the assemblies using the GROMACS msd tool. Avg±SE are shown between all 48 proteins, 24 in each of the two assemblies. *Note, in simulations with $s_{1.06}$ the assemblies start falling apart and the avg diffusion not representative of an in-assembly protein diffusion anymore.

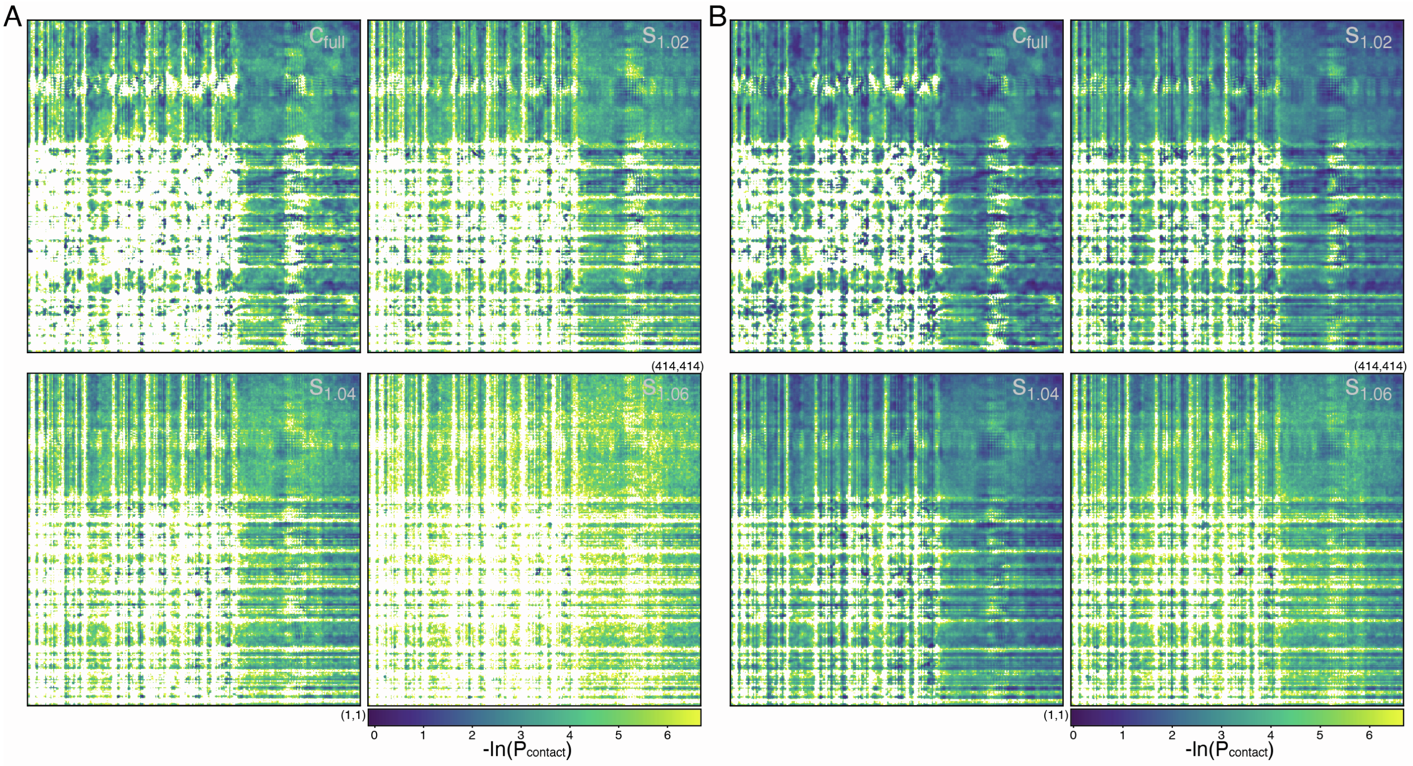

FIGURE S9 Pairwise intermolecular contact map for CG Martini 3 TDP-43 assemblies with scaled protein-water interactions. Residue-residue contact map between all 24 full-length TDP-43 proteins in the simulated assemblies, see Fig. 3A,B and S8. Contact maps are averaged over the last 4 μs of the two assemblies simulated. Here contacts are considered only between backbone atoms and a threshold of 0.8 nm (A) or 1.0 nm (B) was used. Note, the CG HPS-Urry simulations have one-bead-per-residue centered on the C_α atoms and bead diameters are amino acid specific. The distance threshold for the contact analysis (Fig. 3D) is also bead diameters specific and averages to about 0.9 nm (with sidechains implicitly included as change in diameters rather than specifically as additional sidechain beads in Martini), therefore, there is necessarily some difference in the analysis of the different models.

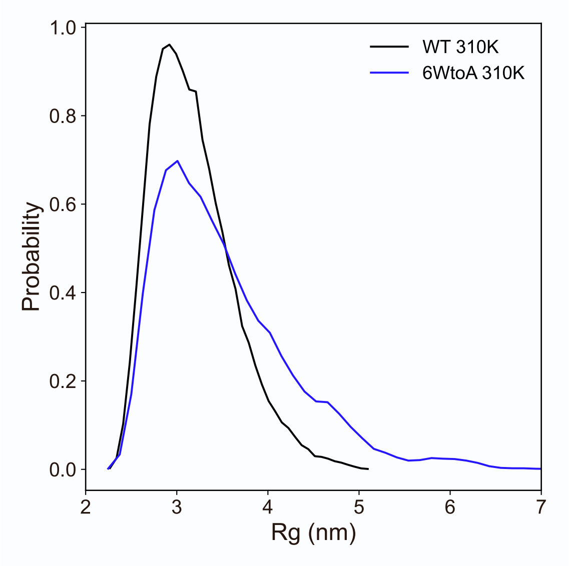

FIGURE S10 Normalized radius of gyration (Rg) distributions of full-length TDP-43 WT and 6WtoA variants from single chain CG HPS-Urry simulations conducted at 310 K.

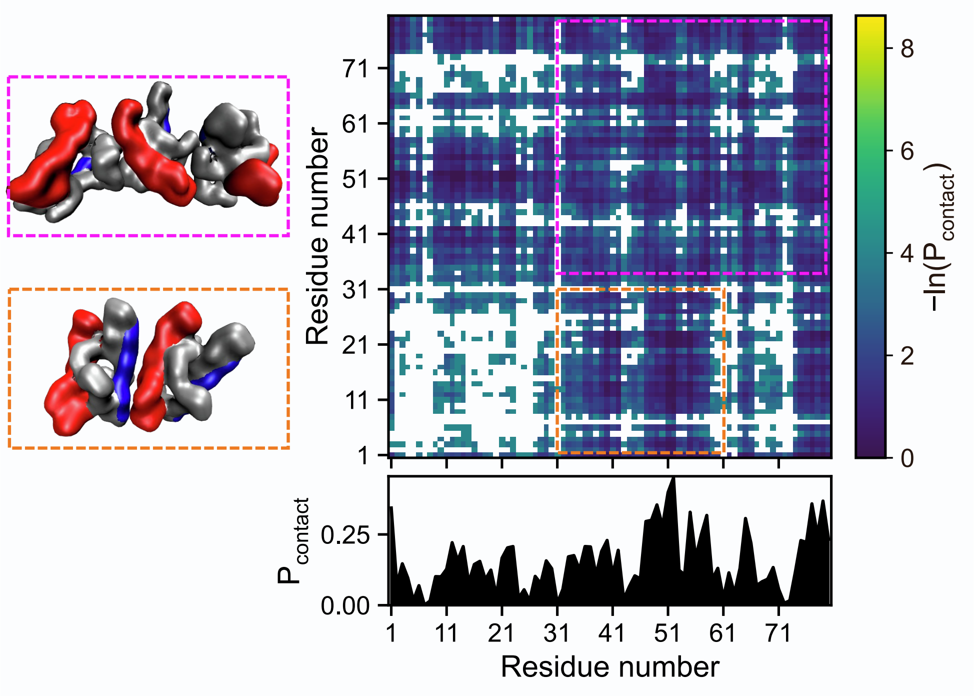

FIGURE S11 Intermolecular contact map of the NTD domain in CG HPS-Urry condensed phase simulations. The contact map reveals the occurrence of site-specific 'head-to-tail' interactions (highlighted in the orange box) by the oppositely-charged regions aa:1-23 (colored as red in the representative snapshots) and aa: 48-54 (colored as blue in the representative snapshots) and non-specific interactions (highlighted in the magenta box, colored as silver in the representative snapshots) that facilitate the oligomerization of NTD-NTD units in the condensed phase.

VIDEO S1 Process of TDP-43 assemblies (in separate file: TDP43-assemblie_cg_simulation.mov). The video shows a Martini 3 simulation of 24 full-length TDP-43 proteins. For each protein the backbone beads are shown in cyan and the N-terminal domain (NTD), the two RNA recognition motifs (RRM1 and RRM2), and C-terminal α-helix are colored in blue, orange, yellow, and purple, respectively. The borders of the simulation box are shown in blue, protein backbone beads in periotic images above and below the box are shown in gray, and ¾ of the water and ions removed for clarity. Initially the 24 proteins are placed apart, they quickly start associating forming larger assemblies, which coalesce into a single assemble. The proteins interact strongly but remain dynamic and solved with significant amount of water and ions (Fig. 3A and B in main text).

SI References

1. Jumper, J., R. Evans, A. Pritzel, T. Green, M. Figurnov, O. Ronneberger, K. Tunyasuvunakool, R. Bates, A. Žídek, A. Potapenko, A. Bridgland, C. Meyer, S. A. A. Kohl, A. J. Ballard, A. Cowie, B. Romera-Paredes, S. Nikolov, R. Jain, J. Adler, T. Back, S. Petersen, D. Reiman, E. Clancy, M. Zielinski, M. Steinegger, M. Pacholska, T. Berghammer, S. Bodenstein, D. Silver, O. Vinyals, A. W. Senior, K. Kavukcuoglu, P. Kohli, and D. Hassabis. 2021. Highly accurate protein structure prediction with AlphaFold. *Nature*. 596(7873):583-589, doi: 10.1038/s41586-021-03819-2, https://doi.org/10.1038/s41586-021-03819-2.
2. Marrink, S. J., H. J. Risselada, S. Yefimov, D. P. Tieleman, and A. H. de Vries. 2007. The MARTINI Force Field: Coarse Grained Model for Biomolecular Simulations. *The Journal of Physical Chemistry B*. 111(27):7812-7824, doi: 10.1021/jp071097f, https://pubs.acs.org/doi/10.1021/jp071097f.
3. Ingólfsson, H. I., C. A. Lopez, J. J. Uusitalo, D. H. de Jong, S. M. Gopal, X. Periole, and S. J. Marrink. 2014. The power of coarse graining in biomolecular simulations. *Wiley Interdisciplinary Reviews: Computational Molecular Science*. 4(3):225-248, doi: 10.1002/wcms.1169.
4. Wright, G. S. A., T. F. Watanabe, K. Amporndanai, S. S. Plotkin, N. R. Cashman, S. V. Antonyuk, and S. S. Hasnain. 2020. Purification and Structural Characterization of Aggregation-Prone Human TDP-43 Involved in Neurodegenerative Diseases. *iScience*. 23(6):101159, doi: https://doi.org/10.1016/j.isci.2020.101159,
